# Successful use of a phage endolysin for treatment of chronic pelvic pain syndrome/chronic bacterial prostatitis

**DOI:** 10.3389/fmed.2023.1238147

**Published:** 2023-08-15

**Authors:** Roy H. Stevens, Hongming Zhang, Michal Kajsik, Rafał Płoski, Malgorzata Rydzanicz, Peter Sabaka, Stanislav Šutovský

**Affiliations:** ^1^Laboratory of Oral Infectious Diseases, Kornberg School of Dentistry, Temple University, Philadelphia, PA, United States; ^2^Department of Bacteriology, Comenius University Science Park, Bratislava, Slovakia; ^3^Department of Molecular Biology, Comenius University Faculty of Natural Sciences, Bratislava, Slovakia; ^4^Department of Medical Genetics, Medical University of Warsaw, Warsaw, Poland; ^5^Department of Infectiology and Geographical Medicine, Faculty of Medicine, Comenius University, Bratislava, Slovakia; ^6^1st Department of Neurology, Faculty of Medicine, Comenius University and University Hospital, Bratislava, Slovakia

**Keywords:** chronic bacterial prostatitis, bacteriophage, endolysin therapy, antibiotic resistance, *Enterococcus faecalis*

## Abstract

Chronic prostatitis (CP) is a common inflammatory condition of the prostate that is estimated to effect 2%–10% of the world’s male population. It can manifest as perineal, suprapubic, or lower back pain and urinary symptoms occurring with either recurrent bacterial infection [chronic bacterial prostatitis (CBP)] or in the absence of evidence of bacterial infection [chronic pelvic pain syndrome (CPPS)]. Here, in the case of a 39 years-old CBP patient, we report the first successful use of a bacteriophage-derived muralytic enzyme (endolysin) to treat and resolve the disease. Bacteriological analysis of the patient’s prostatic secretion and semen samples revealed a chronic *Enterococcus faecalis* prostate infection, supporting a diagnosis of CBP. The patient’s *E. faecalis* strain was resistant to several antibiotics and developed resistance to others during the course of treatment. Previous treatment with multiple courses of antibiotics, bacteriophages, probiotics, and immunologic stimulation had failed to achieve long term eradication of the infection or lasting mitigation of the symptoms. A cloned endolysin gene, encoded by *E. faecalis* bacteriophage *ϕ*Ef11, was expressed, and the resulting gene product was purified to electrophoretic homogeneity. A seven-day course of treatment with the endolysin resulted in the elimination of the *E. faecalis* infection to below culturally detectable levels, and the abatement of symptoms to near normal levels. Furthermore, during the endolysin treatment, the patient experienced no untoward reactions. The present report demonstrates the effectiveness of an endolysin as a novel modality in managing a recalcitrant infection that could not be controlled by conventional antibiotic therapy.

## Introduction

Chronic prostatitis (CP) is an inflammatory condition of the prostate associated with pain and urinary symptoms occurring either with recurrent bacterial infection [chronic bacterial prostatitis (CBP)] or in the absence of evidence of bacterial infection [chronic pelvic pain syndrome (CPPS)] (1–5). CBP may manifest symptoms such as dysuria, localized pain in the perineum, suprapubic region or lower back, and sexual dysfunction including erectile dysfunction and ejaculatory discomfort, along with a positive culture from expressed prostatic secretions (1, 5–8). It is estimated that chronic prostatitis (combined CBP and CPPS) effects approximately 2%–10% of the male population worldwide (1, 2, 9, 10), with a high rate (50%) of recurrence (1, 2, 9). Some studies suggest that 35% to 50% of men are affected by CP at some point in their lives (11). Although infection has not been reported to be associated with most cases of CP (10), when molecular methods (e.g., PCR) were applied diagnostically to cases that were previously determined (by cultural methods) to lack any evidence of bacteriuria or prostate-localized uropathogens, 16S rDNA was detected in prostate biopsies from 77% of these cases, indicating that in fact, most of the CP cases had evidence of bacterial infection, and therefore were in actuality CBP (10, 12).

A wide variety of bacterial species have been isolated from cases of CBP (13). Prominent among these are species of the Gram-negative family, *Enterobacteriaceae* (*Escherichia coli*, *Klebsiella pneumoniae, Proteus mirabilis, Pseudomonas aeruginosa, Acinetobacter* spp., *Citrobacter* spp.) (3, 13), and the Gram-positive Enterococcal genus (*Enterococcus faecalis, Enterococcus faecium*) (3, 7, 13, 14). Enterococcal infections (*E. faecalis* and *E. faecium*) present particularly challenging clinical management problems due to the remarkable hardiness of these species; surviving great extremes in temperature, pH, osmolality as well as starvation and desiccation (15–17). Furthermore, many strains of these species exhibit multidrug resistance (MDR) properties (18–20) such as resistance to *β*-lactams (21, 22), aminoglycosides (23), vancomycin (24), erythromycin (25), tetracycline (26), daptomycin (27), quinupristin-dalfopristin (28) and linezolid (29), complicating management of enterococcal infections.

The emergence and increasing prevalence of MDR bacteria has prompted the search for alternatives to antibiotics to treat these infections. One such promising alternative is the use of bacteriophage (phage) endolysins. Following the recognition that there was a “labile lytic factor in phage lysates” (30), it was proposed that the “lytic factor” (now known to be a muralytic enzyme/endolysin encoded by a phage) could be used to control bacterial infections (31, 32). Consequently, there have been numerous studies on endolysins, detailing their biochemical and biological characteristics, as well as, in some cases, their protective efficacy against infections in *in vivo* animal models [for reviews see references (33–36)]. Finally, one recent study reported the results of a clinical trial testing the efficacy of a phage endolysin (exebacase) in treating *Staphylococcus aureus* (including MRSA) bloodstream infections (BSIs) (37). The results of this clinical trial demonstrated that the combination of the endolysin plus an antibiotic (either semisynthetic penicillins or first-generation cephalosporins for methicillin-sensitive *S. aureus* infections or vancomycin or daptomycin for MRSA) was superior to the antibiotic alone in mitigating the morbidity and mortality of *S. aureus* BSIs.

Previously, the Stevens laboratory isolated a bacteriophage from an infected root canal that infects strains of *E. faecalis* (38). Sequencing and annotation of the phage genomic DNA permitted the identification of a gene that was predicted to code for the phage endolysin (39). Cloning and expression of the putative endolysin gene resulted in the production of a protein whose bacteriolytic activity confirmed the identity of the cloned gene (40). The purified protein [designated open reading frame (ORF) 28 endolysin] was shown to possess multifunctional muralytic activity, acting as an N-acetylmuramidase, an N-acetylglucosaminidase, and an endopeptidase, which could hydrolyze the *E. faecalis* cell wall peptidoglycan (40). The endolysin exhibited remarkably potent lytic activity against many strains of *E. faecalis* including many vancomycin-resistant strains (41). These *in vitro* data suggested the potential for the therapeutic use of the ORF28 endolysin for *E. faecalis* infections. Here we present a case of chronic bacterial prostatitis in which a bacteriophage endolysin was successfully used to treat and mitigate infection and clinical symptoms.

## Methods

### Phage cocktail preparation

Three phages (vB_Efa_VP14, vB_Efa_VP15, and vB_Efa_VP16) prepared in the Science Park Bratislava, were isolated from wastewater samples collected from different wastewater treatment plants in the Bratislava region on a bacterial strain isolated from the patient. Ten milliliters of wastewater, sterilized by passage through a 22 μm filter, was mixed with the same volume of twofold concentrated Trypticase Soy Broth medium and 200 μL of overnight bacterial culture. The inoculated mixture was cultivated overnight at 37°C by shaking. Single phage clones were obtained through three repeated isolations from single plaques on double agar (Supplementary Figure S1). Each of the three clones was purified by precipitation in 10% PEG6000 and 1 M NaCl and subsequent ultracentrifugation in a CsCl gradient. The visible phage band for each phage was collected (~1.5 mLs), and each phage sample was dialyzed against 1 liter of SM buffer (100 mM NaCl; 8 mM MgSO_4_; 50 mM Tris-HCl, pH 7.5; 0.002% gelatin) four times for a minimum of 6 hours each. This yielded 2 mLs of phage with titers of 10^11^–10^12^ PFU/mL. The antimicrobial activity of each of the three phages (as well as cocktails of all three together) against the patient’s *E. faecalis* strain was evaluated by monitoring the optical density (600 nm) of broth cultures of the patient’s *E. faecalis* strain inoculated with the phage. It was found that each of the three phages greatly depressed the growth of the patient’s *E. faecalis* strain, and the cocktail of all three phages completely eliminated bacterial growth (Supplementary Figure S2). The host specificity of individual phages as well as a phage cocktail was determined using a plaque assay on double agar plates. Several bacterial species were tested, but only strains of *E. faecalis* were sensitive. The individual phages were lytic for 32% to 46% of the 28 *E. faecalis* strains tested. The cocktail of all three phages was active against 54% of the *E. faecalis* strains. Supplementary Table S1 contains an overview of the sensitivity of a panel of bacterial strains (including the patient’s strains) to the individual phage isolates. The three phage isolates were sequenced on NextSeq (illumina) using the Nextera protocol. The average coverage was VP14 = 406, VP15 = 639 and VP16 = 542. Phage ends were screened with specific primers and Sanger sequencing. The three phage DNA sequences were deposited in GenBank as accession numbers OR237563 (for phage vB_Efa_VP14), OR237564 (for phage vB_Efa_VP15), and OR237565 (for phage vB_Efa_VP16). These data permitted the assignment of the three newly isolated phages to the genus *Efquatrovirus*. A tree diagram (Supplementary Figure S3) illustrates the relationship between phages vB_Efa_VP14, vB_Efa_VP15, and vB_Efa_VP16, and Efquatrovirus vB_EfaS_AL2 as well as other closely related phages. For cocktail preparation, 1 mL samples were combined to form a three-phage cocktail, each at 10^9^ PFU/mL. The isolated phages were preserved long-term in SM buffer at 6°C for no more than 12 months, however the cocktail batches were prepared monthly.

### Phage endolysin production and purification

The production and purification of the phage endolysin was accomplished as described previously (40). In brief, the gene for the ORF28 endolysin was cloned into a pGEX4T2 expression vector in tandem with a glutathione S-transferase (GST) affinity tag. The recombinant plasmid, also featuring an isopropyl-*β*-D-thiogalactopyranoside (IPTG)-inducible *tac* promotor, was transformed into *E. coli* BL21/DE3. IPTG-induced expression of the linked ORF28 endolysin and GST genes produced an ORF28-GST fusion protein. A sonic extract (SE), made from the induced *E. coli* culture, was applied to a glutathione resin affinity column, and, after nonadsorbed SE material was eluted, the column was extensively washed with buffer to further remove any non-bound material. The ORF28 endolysin-GST fusion protein (bound to the glutathione of the column via the GST) was specifically desorbed from the column by the addition of a buffer containing glutathione. The process was repeated 4 times until only two protein bands (representing the ORF28-GST fusion protein and the GST protein alone) could be seen by SDS-PAGE analysis of the desorbed material. The ORF28 endolysin protein was recovered from the ORF28-GST fusion protein by reapplying the purified fusion protein to the affinity column and digesting the bound fusion protein with thrombin to cleave the thrombin-sensitive linkage between the ORF28 endolysin and the GST protein. The liberated ORF28 protein was then eluted from the column and collected. The homogeneity of the affinity-purified ORF28 protein was confirmed by SDS-PAGE analysis. As a final step in the purification process, the electrophoretically-homogeneous ORF28 endolysin preparation was passed through a 0.22 μ pore size sterilizing filter. The final purified endolysin preparation had a protein concentration of 0.8 mg/mL.

### Spot testing endolysin activity

Spot testing was used to examine the activity of the ORF28 endolysin against the *E. faecalis* strain isolated from the patient. 0.1 mL of an overnight culture of *E. faecalis* strain 587A, originally isolated by a clinical laboratory in Bratislava in June 2020, was grown in brain heart infusion (BHI) Broth and was inoculated into 3 mL of molten soft agar (BHI broth containing 0.7% agar). This was poured into plates over a layer of BHI agar (1.5%) and allowed to solidify and dry for approximately 15 min. Drops (3 μL) of dilutions of an ORF28 endolysin suspension were then applied to the surface of the solidified soft agar layer. The drops were allowed to dry into the soft agar layer, and the plates incubated overnight at 37°C. The plates were then examined for clear zones where the drops were originally placed, indicating the lytic activity of the endolysin against the *E. faecalis* strain.

### Whole exome sequencing analysis

(Department of Medical Genetics, Medical University of Warsaw, Poland). A library was prepared using the Human Core Exome Kit (Twist Bioscience, South San Francisco, CA, United States), according to manufacturer’s instruction, and paired-end sequenced (2 × 100 bp) on a NovaSeq 6000 platform (Illumina, San Diego, CA, United States). Bioinformatic analysis of raw whole exome sequencing (WES) data and variants prioritization were performed as previously described (42).

## Results

### Case description

**Figure 1:**
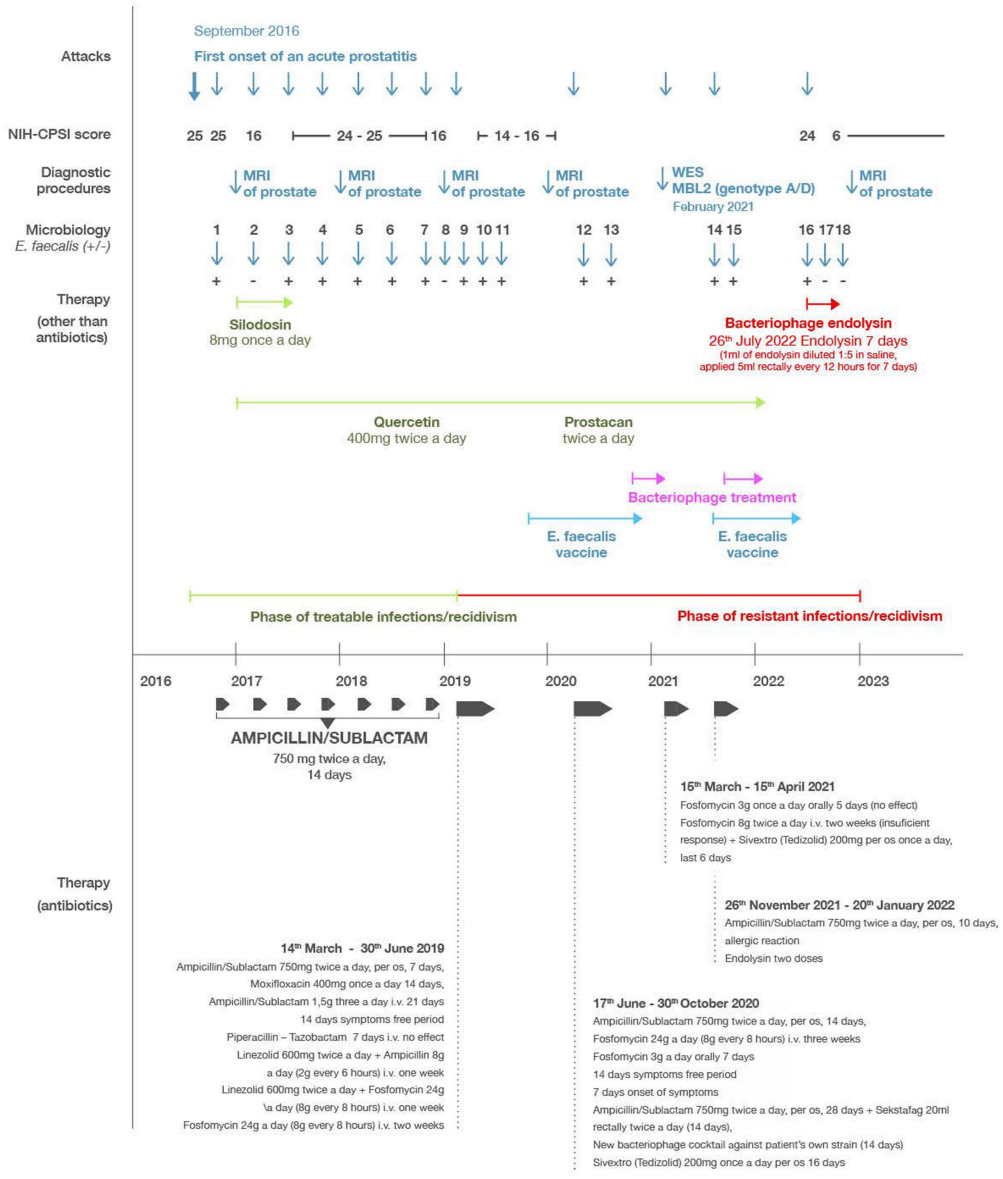
Timeline of the most relevant attacks, diagnostic procedures, microbiological results (dark blue), antibiotic therapies (dark grey), *Enterococcus faecalis* vaccination (light blue), phage therapy (magenta), endolysin therapy (red), and other supportive therapies (green). “Microbiology” refers to microbiological analysis determining the presence (*E. faecalis*^+^) or absence (*E. faecalis*^**−**^) of detectable *E. faecalis* infection.

A 39 years-old Slovakian man was referred to a neurologist in October 2016 after 2 months of neuropathic pain in the perineum, which radiated to the scrotum and the entire anogenital area. The pain developed following repeated cold stimuli in the fall of 2016. The patient described several types of pain: pain in the perineum was dominant, radiating to the rectum, scrotum, and penis. Pain behind the pubic bone was also present. Initially, the pain was paroxysmal and neuralgiform, later it was continuous. Objective neurological findings included significant hyperalgesia of the entire anogenital area. The condition was concluded as chronic pelvic pain syndrome. The patient underwent a battery of examinations aimed at clarifying the origin of the pain. A urologist found an enlarged and painful prostate. Ultrasonography and subsequent magnetic resonance imaging confirmed prostatitis. Cultured ejaculate and expressed prostatic secretions repeatedly confirmed an infection by *Enterococcus faecalis* with high sensitivity to ampicillin/sulbactam (Figure 1, Micro 1). At that time, according to the NIH chronic prostatitis symptom index (NIH-CPSI) (43), the patient reported a pain score of 11 out of 21, a urinary symptom score of 5 out of 10, a quality-of-life impact score of 9 out of 12, and a total score 25, with higher scores indicative of worse outcomes. (In comparison, mean scores for pain, urinary symptoms and quality of life from a cohort of CBP patients were 8.7 ± 5.7, 4.1 ± 3.1, and 6.7 ± 3.6 respectively). The severity of the CBP can be classified as mild (0–9 points), moderate (10-18points) or severe (19–31 points) according to the NIH-CPSI score (43).

The patient’s course of treatment is illustrated in the timeline shown in Figure 1. Initial treatment consisted of ampicillin/sulbactam for 2 weeks with satisfactory clinical improvement and bacteriological eradication of enterococcus (Figure 1, Micro 2). However, all the clinical symptoms previously described persisted but at milder intensity. The average NIH-CPSI score after this treatment was 16. Over the next 3 years, the patient had at least three exacerbations per year, when the NIH-CPSI score reached 26 or more, and the presence of *E. faecalis* was always confirmed during flares (Figure 1, Micro 3–7). After treatment with ampicillin/sulbactam or quinolones for 2 weeks, there was a clinical improvement and bacteriological negativity (Figure 1, Micro 8). The NIH-CPSI score averaged 16 during remission stages. In addition to the antibiotics, supplementary therapy consisting of silodosin 4 mg once a day, serenoa rapens, and quercetin 400 mg twice a day, was added to the patient’s treatment, however, this produced no significant effect on relieving the symptoms.

In March 2019, prostatitis recurred, coincident with a positive culture of *E. faecalis*, which showed good sensitivity to ampicillin/sulbactam (MIC mg/L AMP 1, VAN 2, CIP 0.5 U, TET ≥16, GEN 128, Figure 1, Micro 9). The patient was re-treated with ampicillin/sulbactam in the usual dose, but after a week of therapy the symptoms did not subside. Ampicillin/sulbactam was changed to moxifloxacin, which showed equally good sensitivity *in vitro*. After 14 days of treatment, tachycardia and severe headaches occurred and moxifloxacin was discontinued. After 3 days of withdrawal, the difficulties returned completely. Re-culture of ejaculate and expressed prostatic secretion again showed *E. faecalis* with practically the same MIC values (Figure 1, Micro 10). A 21 days course of ampicillin/sulbactam (4.5 g/day, i.v.) was initiated, resulting in complete abatement of symptoms. After 3 weeks of withdrawal, the difficulties returned again. The culture of the ejaculate and prostatic secretion conducted for the third time after massage again showed *E. faecalis*, but with higher MIC values (AMP 2, VAN 4, CIP 1 U, TET ≥16, GEN 128, Figure 1, Micro 11).

Based on these results, the patient was placed on long-term treatment with high doses of antibiotics consisting of linezolid 600 mg twice a day in combination with ampicillin 2 g every 6 h for the first week. In the second week, the linezolid (600 mg twice a day) was combined with fosfomycin 8 g every 8 h. For the third and fourth weeks, he received monotherapy with fosfomycin 8 g every 8 h. After this treatment, the disease subsided and did not recur for a year. During this period the patient underwent treatment by enterococcal vaccine from his own strain of enterococcus.

In June 2020, there was another recurrence of the disease in the sense of urgency and pelvic pain. Culture of the ejaculate and prostatic secretion after the massage again showed *E. faecalis* with an MIC of AMP 2. Furthermore, the clinical microbiology laboratory reported that the *E. faecalis* isolate was resistant to a variety of antibiotics including oxacillin, cefoxitin, gentamicin and tetracycline (Figure 1, Micro 12). The patient began treatment with ampicillin/sulbactam 750 mg twice a day. After a temporary improvement, the condition worsened from the fifth day of treatment. Due to intolerance to quinolones and the previous eradication of the pathogen with fosfomycin, therapy continued with fosfomycin 8 g three times a day intravenously for 21 days, followed by fosfomycin 3 g per day orally for 7 days, i.e., a total of 28 days. After this treatment, the difficulties subsided. After 2 weeks, however, the difficulties recurred and again, enterococcus was cultivated from ejaculate (Figure 1, Micro 13).

### Application of phage therapy

Considering that the patient received the highest possible doses of fosfomycin for 21 days, and was intolerant to quinolones, we proceeded to a combination of bacteriophages (Sekstafag) 20 mL rectally twice a day and ampicillin/sulbactam 750 mg twice a day *per os* for 2 weeks, without any clinical effect. Furthermore, a new bacteriophage cocktail against patient’s own strain in a concentration of 10^9^ pfu/mL, with convincing *in vitro* activity, had been prepared in the Science Park Bratislava. The cocktail contained three newly isolated phages (vB_Efa_VP14, vB_Efa_VP15, vB_Efa_VP16) belonging to the genus *Efquatrovirus*. Each phage had a unique host specificity and efficiently lysed the patient’s strain. The patient used this cocktail for the next 2 weeks, 10 mL twice a day applied rectally, however no clinical effect was observed. In September 2020, the patient started taking linezolid 600 mg twice a day for 21 days.

### Application of phage endolysin eliminates enterococcal infection and mitigates CBP symptoms

**Figure 2:**
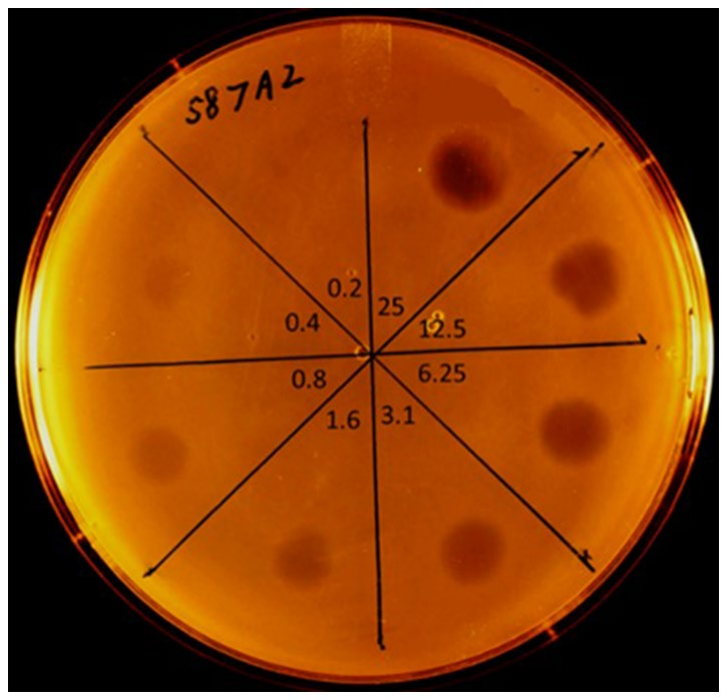
Spot testing sensitivity of *E. faecalis* strain (587A2) from the CBP patient to purified phage ORF28 endolysin. Dilutions of purified endolysin (original concentration = 800 μg/mL) spotted onto a soft-agar lawn of *E. faecalis* 587A2. Lytic zones observed after overnight incubation @ 37°C. Numbers towards center of plate indicate the concentration (μg/mL) of ORF28 applied in each section of the plate. Lytic zone observable down to endolysin concentration of 0.4 μg/mL.

The lack of improvement of the patient’s condition prompted a search for an alternative therapy to the antibiotic treatments previously employed. In September 2020 a request was sent from Slovakia to the Stevens laboratory at Temple University, Philadelphia, for an *E. faecalis*-specific bacteriophage or a phage-based lytic enzyme that could be capable of degrading an *E. faecalis* biofilm. The laboratory had both: previously, a genetically engineered derivative of *E. faecalis* phage (*ϕ*Ef11) had proven in *in vitro* testing to infect many strains of *E. faecalis*, reduce the populations of *E. faecalis* cultures, and drastically disrupt *E. faecalis* biofilms (44, 45). However, the genetically modified phage [*ϕ*Ef11/FL1C(Δ36)*P*^nisA^] possessed a nisin-dependent promoter (*P*^nisA^) that required the presence of nisin, as a cofactor for activation. While this was advantageous for controlling phage activity in *in vitro* experimental conditions, it would not be suitable for *in vivo* clinical application. However, the *ϕ*Ef11 phage endolysin (ORF28 endolysin) was available from the laboratory and had previously exhibited rapid and profound lysis of cells of most *E. faecalis* strains, including those that were antibiotic-resistant (40, 41). The patient’s *E. faecalis* strain was isolated and sent to the Stevens laboratory for sensitivity testing against the *ϕ*Ef11 phage ORF28 endolysin. Spot testing of dilutions of the purified endolysin on lawns of the patient’s *E. faecalis* isolate revealed that this strain was indeed extremely sensitive to the lytic action of the endolysin (Figure 2).

**Figure 3:**
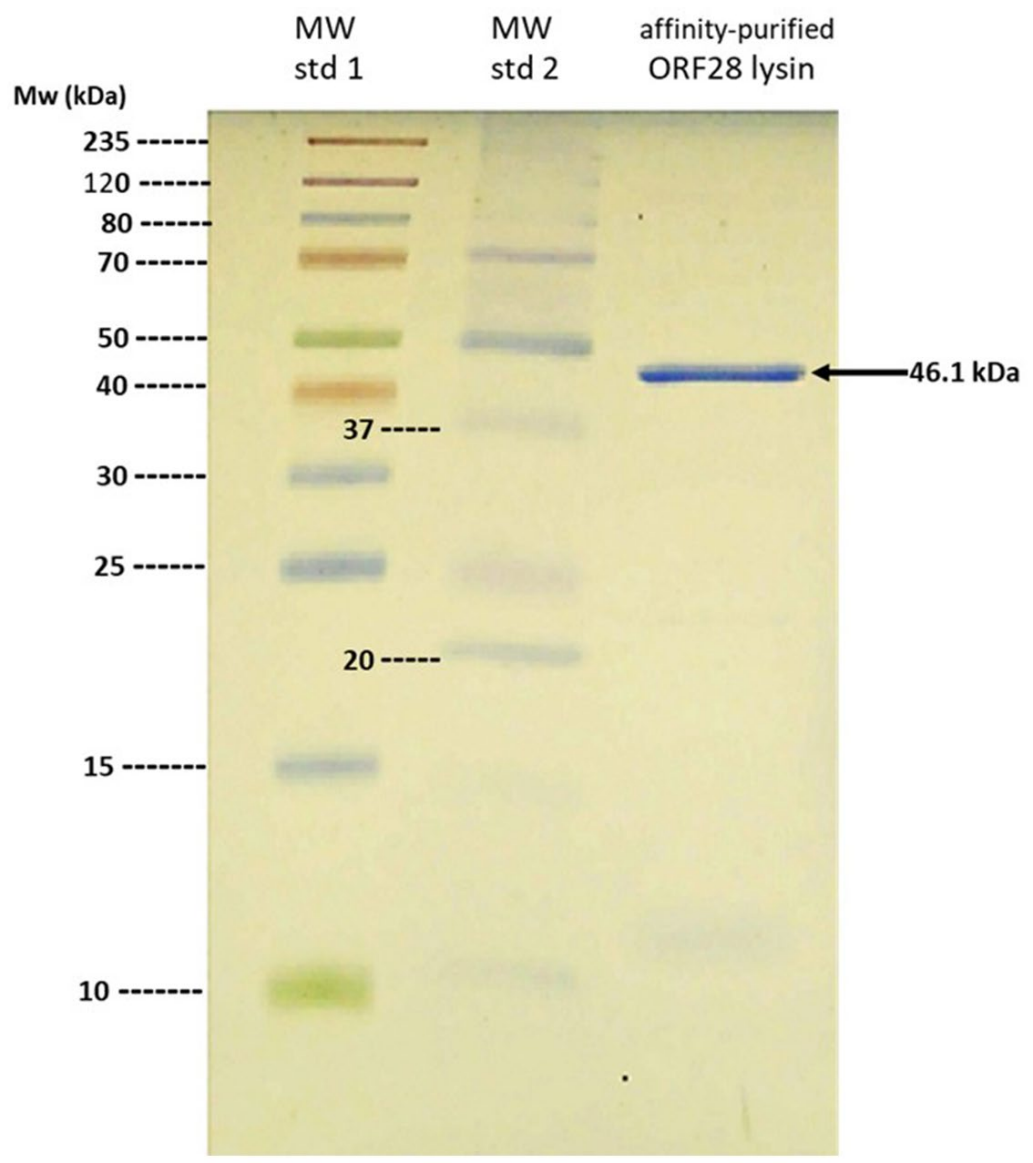
SDS-PAGE analysis of purified ORF28 endolysin. Note single 46.1 kDa band from affinity-purified ORF28 endolysin.

Considering the pronounced endolysin-sensitivity of the patient’s *E. faecalis* strain, it was not unreasonable to entertain the possibility that the endolysin might have a beneficial effect in controlling the patient’s infection. In this regard, Temple University’s Institutional Review Board was contacted to ensure that the contemplated endolysin therapy would be consistent with local (U.S.) regulatory requirements. Regulatory requirements in Slovakia consisted of the recognition that (1) bacteriophages and their products are considered as an alternative to antibiotics and (2) the clinical use of bacteriophages and their products is solely governed by the expert opinion of the attending physician or consultant. Consequently, the expert opinion of an infectious diseases specialist was obtained, concluding that the patient was suffering from “…a biofilm infection which can no longer be eradicated with current antibiotics”, and that “…therapy with bacteriophage lysine (*sic*) as a last resort in the treatment of refractory bacterial prostatitis.” Therefore, with no additional regulatory requirements to be satisfied, purification of the phage endolysin was completed, resulting in the isolation of an electrophoretically homogeneous protein with a molecular mass of 46.1 kDa, which is the predicted size of the phage ORF28 endolysin (Figure 3). The patient was fully informed about potential risks and benefits of the treatment and signed an informed consent and approved the publication of his course of treatment.

The sterile, purified endolysin preparation was sent to Slovakia in November of 2020 however at that time, the patient was already asymptomatic due to linezolid therapy. During the year 2021 the patient experienced several attacks of prostatitis usually treated with ampicillin/sulbactam or levofloxacin. Furthermore in 2021, the patient underwent whole exome sequencing (WES) analysis in the Department of Medical Genetics, Medical University of Warsaw, Poland. WES revealed a heterozygous variant in *MBL2* gene (hg38, chr10:g.052771482-G > A, NM_000242.3: c.154C > T/p.(Arg52Cys), rs5030737), which refers to D-allele and HYD haplotype (patient’s genotype A/D and haplotype HYA/HYD). The p.(Arg52Cys) variant is described as “pathogenic” according to ClinVar database (Accession: RCV000015426.29) in relation to mannose-binding lectin (MBL) deficiency (MIM#614372). Subsequent immunological examination showed a reduced level of MBL to 375 ng/mL (normal value more than 2,880).

By October/November of 2021, the prostatitis worsened, with more intense symptoms in terms of urgencies, nycturia and pelvic pain, and culture of the ejaculate again confirmed *E. faecalis* (Figure 1, Micro 14, 15). For this, the patient began applying probiotics consisting of lactobacilli and the above-mentioned bacteriophage cocktail (10^9^ pfu/mL), which he continued for the next 2 weeks, 20 mL rectally twice a day, however the symptoms continued to worsen. The patient was then again put on ampicillin-sulbactam (750 mg twice a day orally) and the condition began to improve, however after 4 days, the patient experienced a severe allergic reaction consisting of whole body itching and exanthema. This necessitated the discontinuation of the ampicillin/sulbactam treatment and its replacement by fosfomycin 3 g once a day orally. Again, a similar severe allergic reaction (whole body itching and exanthema) ensued, and the fosfomycin also had to be discontinued. At this point the patient applied the phage endolysin preparation, which had been stored refrigerated since its arrival (prior laboratory studies demonstrated that the endolysin was extremely stable, and could retain its activity for several years, if kept refrigerated) (40). The preparation was diluted 1:5 in saline, and two doses of 5 mL were applied rectally 12 h apart. After the administration of the two doses, the symptoms subsided, but the patient did not have additional doses available, so he could not continue the endolysin therapy.

In July 2022 there was a significant recurrence, and culture of the ejaculate again confirmed *E. faecalis* (Figure 1, Micro16). The total NIH-CPSI score at this point was 24 (a pain score of 7, a urinary symptom score of 8, and a quality-of-life impact score of 9). Additional endolysin received from the Stevens laboratory was immediately applied in the same dose (1 mL of endolysin diluted 1:5 in saline and applied 5 mL rectally every 12 h for 7 days) with significant clinical improvement and bacteriological eradication of enterococcus. The total NIH-CPSI score at this point was 6 (a pain score of 0, a urinary symptom score of 3, and a quality-of-life impact score of 3). After completing the treatment in August of 2022, the culture of the ejaculate was sterile, and the expressed prostatic secretion contained only coagulase-negative staphylococci (Figure 1, Micro 17). The patient reported no untoward reactions during or after the endolysin treatment with any of the administrations. From this period (August 9, 2022) until the last check-up (February 9, 2023), the patient did not have a recurrence of bacterial prostatitis. His CPS score remained at 6 points (a pain score of 0, a urinary symptom score of 3, and a quality-of-life impact score of 3). His most recent bacteriological analysis, in November of 2022 was negative (Figure 1, Micro 18) and MRI of the prostate from December 2022 showed consolidation of inflammatory changes of the prostate by 30% compared to an examination conducted in September 2020. The patient is still taking quercetin 400 mg once a day and Prostacan (*Serenoa rapens* and *Urtica dioica* extract) once a day.

## Discussion

Chronic prostatitis is a chronic recurrent disease with a very complex etiology and pathogenesis, which significantly reduces the quality of life of patients. Predisposing factors are usually hidden immunological deficits, which are manifested by increased susceptibility of the urogenital tract to bacterial invasion (46–48). Numerous recent studies have reported that *E. faecalis* is either the first (49–53) or second (54–57) most frequently isolated organism from CBP cases. *E. faecalis* frequently forms a biofilm in acini or around prostatic calculi, which protects it from the impact of antibiotics (58). Repeated administration of antibiotics is associated with a high risk of the development of resistance, and cumulative toxic effects and allergic reactions. Therefore, the search for alternative approaches is the right way forward.

Here, we present the case of a 39 years-old man with chronic pelvic pain due to CBP. This report points to the torpidity and the chronic relapsing nature of the disease, and the failure of antibiotic therapy. Diagnostically, WES revealed an MBL2 gene polymorphism with genotype A/D instead of wild type AA and subsequent immunologic testing confirmed MBL2 deficiency. Considering this, several alternative treatment options were attempted. From the history of the present case, the following can be stated: bacteriophage cocktails were not effective. Supportive treatment, in the form of phytopharmaceuticals (*Serenoa rapens*, *Urtica dioica* and quercetin extracts), microbiome modification via probiotics, and administration of an autovaccine developed from the patient’s own *E. faecalis* strain, all failed to prevent disease reoccurrence and continued infection. We perceive the most significant impact on the course of the disease was due to the application of bacteriophage endolysin rectally, which resulted in the elimination of enterococcus and a long-term asymptomatic period.

Antibiotic therapy is the conventional standard of care for treatment of chronic bacterial prostatitis (4, 59). In the present case, conventional therapy using antibiotics, was ineffective due to the development of resistance to many antibiotics by the *E. faecalis* strain causing the infection, and the intolerably severe allergic reactions caused by the antibiotics that were used. In addition, biofilms that can form in the infected prostate (60, 61), can be relatively resistant to the antimicrobial effects of antibiotics (62).

Phage therapy has been proposed as a useful alternative to antibiotics. In this regard, several studies have reported the successful use of phage therapy in treating cases of CBP (63–66). A personalized bacteriophage cocktail that was active (*in vitro*) against the patient’s own strain of *E. faecalis* was used in treating the patient’s infection. In light of the prior reports of successful phage therapy in treating chronic bacterial prostatitis, it is not clear why this was not effective in the present case.

This report demonstrates the utility and efficacy of bacteriophage endolysin therapy in treating recalcitrant infections; particularly those due to drug-resistant bacteria or in cases where effective antibiotic treatment is precluded due to serious adverse reactions. The efficacy of the ORF28 endolysin for controlling this patient’s *E. faecalis* infection was presumably due to the ability of the endolysin to lyse and kill the infecting organisms. Previous *in vitro* data demonsterated that the ORF28 endolysin caused rapid and profound lysis of sensitive *E. faecalis* strains (40), and that the patient’s *E. faecalis* strain was shown to be sensitive to the endolysin. Thus, it was not unreasonable to anticipate a beneficial effect of the endolysin on the patient’s *E. faecalis*-infected prostate, provided that the endolysin could gain access to the infected prostate. Anatomic studies disclose that the rectal venous plexus/hemorrhoidal plexus communicates with the prostatic venous plexus via the vesical venous plexus (67). This could provide an entrée for rectally-applied endolysin to the prostate. In animal studies involving mice and rabbits, rectally-applied bacteriophages could be detected in the circulation within minutes (68). Therefore, the positive outcome that we observed is consistant with both the activity of the phage endolysin, and its potential availability to the site of infection.

## Data availability statement

The datasets presented in this study can be found in online repositories. The names of the repository/repositories and accession number(s) can be found at: https://www.ncbi.nlm.nih.gov/genbank/, GQ452243.

## Ethics statement

The studies involving humans were approved by Institutional Review Board, Temple University. The studies were conducted in accordance with the local legislation and institutional requirements. The participants provided their written informed consent to participate in this study. Written informed consent was obtained from the individual(s) for the publication of any potentially identifiable images or data included in this article.

## Author contributions

RS: conceptualized the project, isolated and characterized the bacteriophage from which the endolysin was obtained, and provided the genetic information for cloning the endolysin gene. HZ: cloned the endolysin gene and purified the endolysin. RS and HZ: characterized the endolysin. PS: provided infectious disease expertise and expert opinion authorizing the compassionate use of the endolysin. MK: isolated and characterized the bacteriophages used for phage therapy. RP and MR: oversaw the whole exosome sequencing and genomic data analysis. SŠ: oversaw patient management and all clinical aspects of the study. RS and SŠ: wrote the manuscript. All authors contributed to the article and approved the submitted version.

## Conflict of interest

The authors declare that the research was conducted in the absence of any commercial or financial relationships that could be construed as a potential conflict of interest.

## Publisher’s note

All claims expressed in this article are solely those of the authors and do not necessarily represent those of their affiliated organizations, or those of the publisher, the editors and the reviewers. Any product that may be evaluated in this article, or claim that may be made by its manufacturer, is not guaranteed or endorsed by the publisher.
