# Supplementary material for: Successful use of a phage endolysin for treatment of chronic pelvic pain syndrome/chronic bacterial prostatitis
Source: Front Med (Lausanne). 2023 Aug 15;10:1238147. doi: 10.3389/fmed.2023.1238147 (PMC10462781; doi:10.3389/fmed.2023.1238147)
Supplement: Supplementary file 1 [file Table_1.pdf]

Table S1. Host Range of phages used in cocktail. The specificity of individual phages as well as the cocktail of all three phages was determined using plaque assays on double agar plates. Several species were tested, however only *E. faecalis* strains were sensitive.

Bacterial strain	Cultivated from	vB_Efa_VP14	vB_Efa_VP15	vB_Efa_VP16	Phage cocktail
Enterococcus faecalis 587A Patient's strain (2020-June)	expressed prostatic fluid	lysis	lysis	lysis	lysis
Enterococcus faecalis 8041B Patient's strain (2021-10-01)	uretra	lysis	lysis	lysis	lysis
Enterococcus faecalis 9844A Patient's strain (2021-11-30)	ejaculate	lysis	lysis	lysis	lysis
Enterococcus faecalis 11883A	wound infection	lysis	lysis	lysis	lysis
Enterococcus faecalis 987A	wound infection	N	N	lysis	lysis
Enterococcus faecalis 13555B	wound infection	lysis	lysis	lysis	lysis
Enterococcus faecalis 14397C	diabetic foot ulcer infection	lysis	N	N	lysis
Enterococcus faecalis 3177A	diabetic foot ulcer infection	lysis	lysis	N	lysis
Enterococcus faecalis 26823B	diabetic foot ulcer infection	lysis	lysis	N	lysis
Enterococcus faecalis 27553B	diabetic foot ulcer infection	N	N	N	N
Enterococcus faecalis 27411A	diabetic foot ulcer infection	N	N	N	N
Enterococcus faecalis 34843B	diabetic foot ulcer infection	N	N	N	N
Enterococcus faecalis 33257E	gangraena pedis diabetica	N	N	N	N
Enterococcus faecalis 2905 B	gangraena pedis diabetica	N	N	N	N
Enterococcus faecalis 3764 A	gangraena pedis diabetica	lysis	lysis	lysis	lysis
Enterococcus faecalis 4224	diabetic foot ulcer infection	N	N	lysis	lysis
Enterococcus faecalis 1859	wound infection	N	N	N	N
Enterococcus faecalis 7423	wound infection	N	N	N	N
Enterococcus faecalis 3771A	gangraena pedis diabetica	N	N	N	N
Enterococcus faecalis 3117A	wound infection	N	N	N	N
Enterococcus faecalis 26375D	wound infection	N	N	N	N
Enterococcus faecalis 3413	wound infection	N	N	N	N
Enterococcus faecalis 28063A	gangraena pedis diabetica	lysis	lysis	lysis	lysis
Enterococcus faecalis 34845A	gangraena pedis diabetica	lysis	lysis	N	lysis
Enterococcus faecalis 25668B	diabetic foot ulcer infection	lysis	lysis	N	lysis
Enterococcus faecalis 30689A	gangraena pedis diabetica	lysis	lysis	N	lysis
Enterococcus faecalis 30687B	diabetic foot ulcer infection	N	N	N	N
Enterococcus faecalis 21029A	diabetic foot ulcer infection	N	N	N	N
Staphylococcus aureus 31859/4	diabetic foot ulcer infection	N	N	N	N
Staphylococcus aureus 18 741B	gangraena pedis diabetica	N	N	N	N
Staphylococcus aureus 1861 A	diabetic foot ulcer infection	N	N	N	N
Enterobacter cloacae 415	gangraena pedis diabetica	N	N	N	N
Enterobacter cloacae 33 249A	gangraena pedis diabetica	N	N	N	N
