# Supplementary figures and images for: Successful use of a phage endolysin for treatment of chronic pelvic pain syndrome/chronic bacterial prostatitis
Source: Front Med (Lausanne). 2023 Aug 15;10:1238147. doi: 10.3389/fmed.2023.1238147 (PMC10462781; doi:10.3389/fmed.2023.1238147)

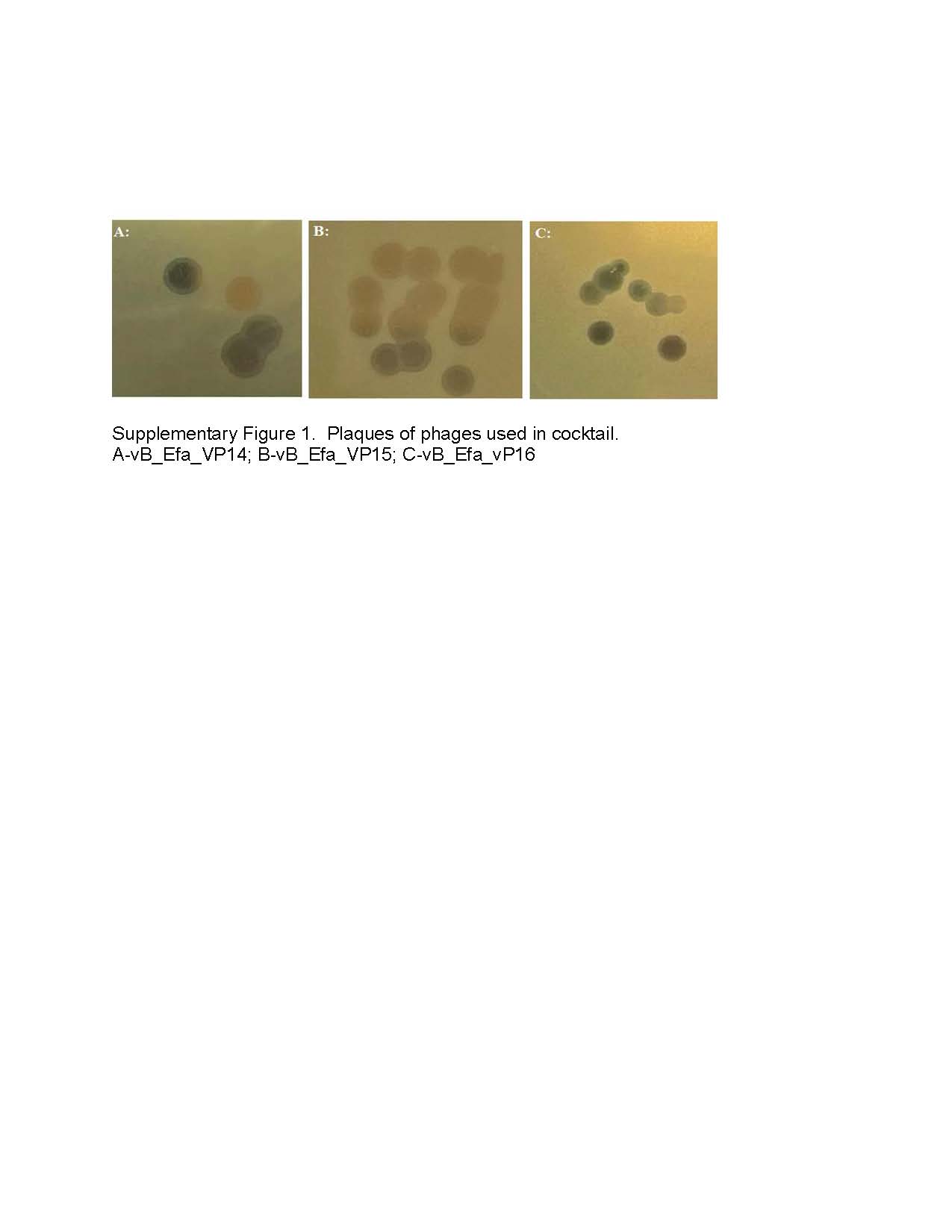

Supplement: Supplementary file 2 [file Image_1.JPEG]

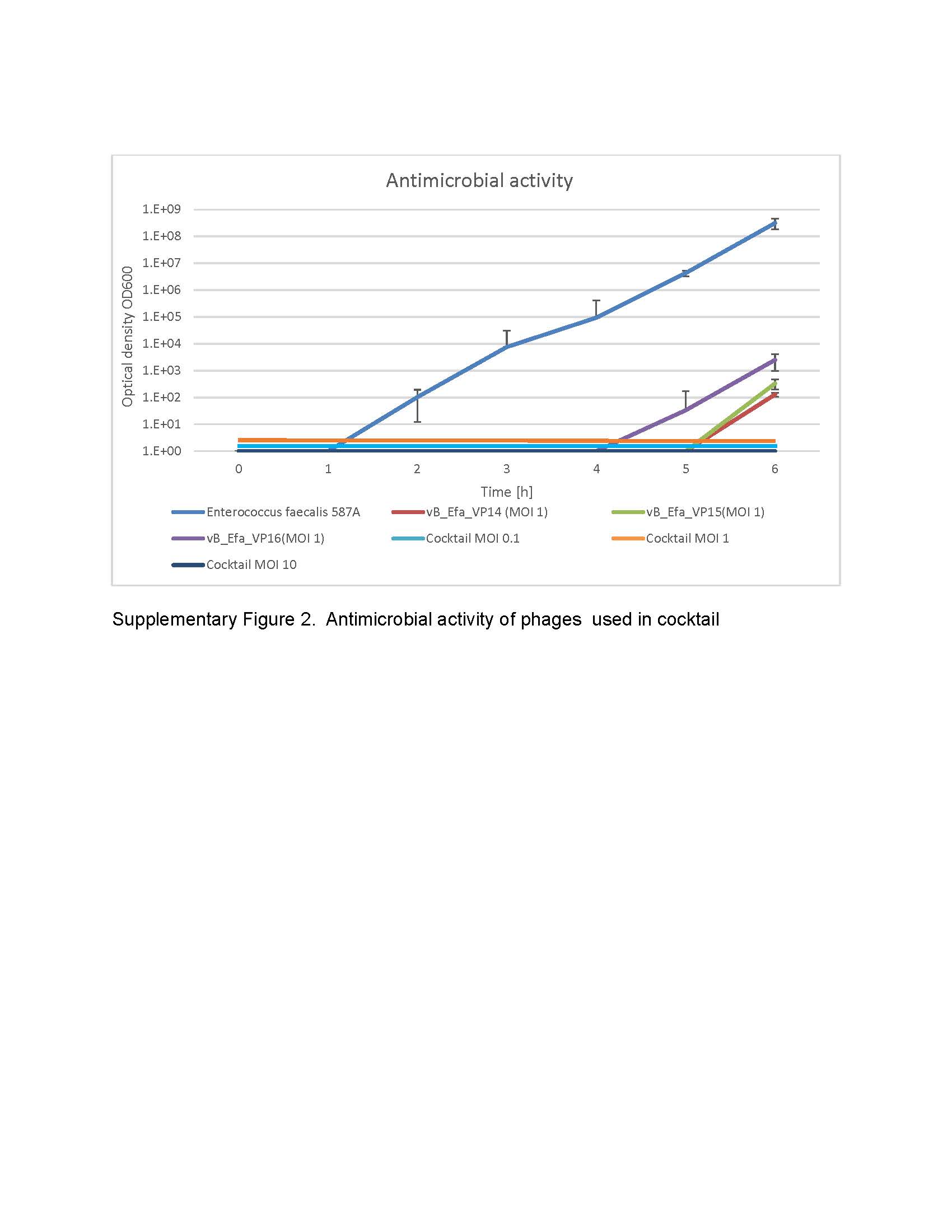

Supplement: Supplementary file 3 [file Image_2.JPEG]

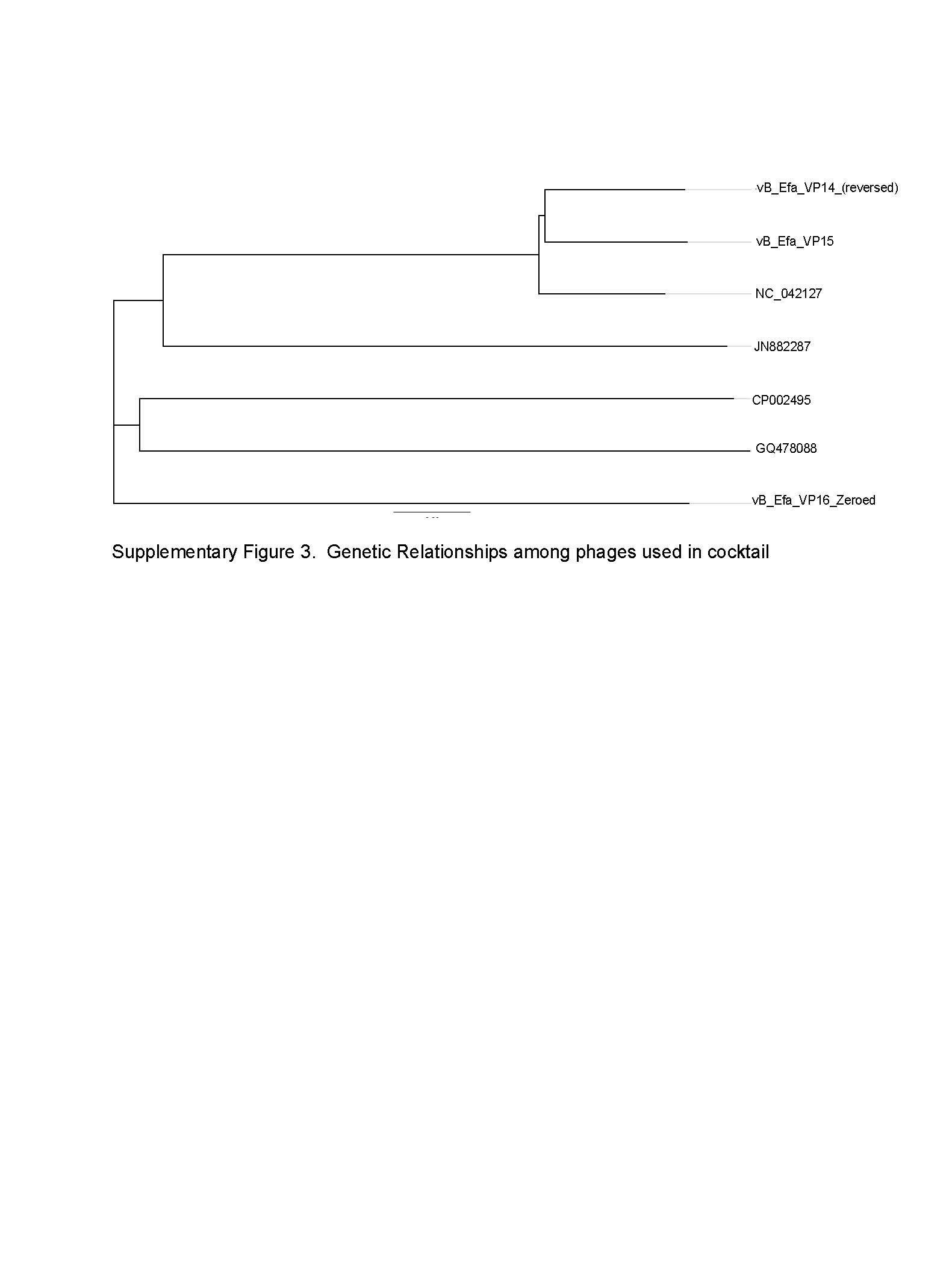

Supplement: Supplementary file 4 [file Image_3.JPEG]
